# A Behavior-Based Model to Validate Electronic Systems Designed to Collect Patient-Reported Outcomes: Model Development and Application

**DOI:** 10.2196/56370

**Published:** 2024-09-17

**Authors:** Sultan Attamimi, Zoe Marshman, Christopher Deery, Stephen Radley, Fiona Gilchrist

**Affiliations:** 1 Academic Unit of Oral Health Dentistry and Society University of Sheffield Sheffield United Kingdom; 2 Department of Preventive Dentistry College of Dentistry University of Hail Hail Saudi Arabia; 3 Obstetrics and Gynaecology Unit, Jessop Wing Sheffield Teaching Hospital Sheffield United Kingdom

**Keywords:** patient-reported outcome, PRO, electronic PRO, user acceptance testing, system validation, patient-reported outcomes, electronic PROs, user acceptance, validation model, paediatric dentistry

## Abstract

**Background:**

The merits of technology have been adopted in capturing patient-reported outcomes (PROs) by incorporating PROs into electronic systems. Following the development of an electronic system, evaluation of system performance is crucial to ensuring the collection of meaningful data. In contemporary PRO literature, electronic system validation is overlooked, and evidence on validation methods is lacking.

**Objective:**

This study aims to introduce a generalized concept to guide electronic patient-reported outcome (ePRO) providers in planning for system-specific validation methods.

**Methods:**

Since electronic systems are essentially products of software engineering endeavors, electronic systems used to collect PRO should be viewed from a computer science perspective with consideration to the health care environment. On this basis, a testing model was blueprinted and applied to a newly developed ePRO system designed for clinical use in pediatric dentistry (electronic Personal Assessment Questionnaire-Paediatric Dentistry) to investigate its thoroughness.

**Results:**

A behavior-based model of ePRO system validation was developed based on the principles of user acceptance testing and patient-centered care. The model allows systematic inspection of system specifications and identification of technical errors through simulated positive and negative usage pathways in open and closed environments. The model was able to detect 15 positive errors with 1 unfavorable response when applied to electronic Personal Assessment Questionnaire-Paediatric Dentistry system testing.

**Conclusions:**

The application of the behavior-based model to a newly developed ePRO system showed a high ability for technical error detection in a systematic fashion. The proposed model will increase confidence in the validity of ePRO systems as data collection tools in future research and clinical practice.

## Introduction

The focus of health care systems has shifted fundamentally by placing patients at the center of care to promote improved service satisfaction and better treatment outcomes. The shift is described in the UK National Health Service initiatives and by research funding bodies [1,2]. This trend is reflected in the growing use of patient-reported outcomes (PROs), defined as “any report of the status of a patient’s health condition that comes directly from the patient without interpretation of the patient’s response by a clinician or anyone else” [3]. PRO measures (PROMs) are tools used to capture PRO in the form of questionnaires with different constructs and measurement schemes. PROMs have been increasingly used for service evaluation and quality measurement and have been embraced in routine clinical care [4]. The traditional administration method of PROMs in a paper format can be a burden on clinicians and researchers, which makes a remote and automated method of collection potentially beneficial [5].

Electronic PRO (ePRO) is an innovation in which participants have expressed positive thoughts and attitudes [5]. Many systems have been developed to collect PRO electronically [6-8]. Guidance on system design and methods to preserve the integrity of the PROMs psychometric properties during electronic conversion is available [9-11]. The involvement of patients as end users in investigating the usability of ePRO has been explored [9,12]. Recommendations on testing systems designed to collect clinical outcomes are outlined by the PRO Consortium [13]. Evidence is lacking on a generalized model to inform appropriate system-specific validation methods of the ePRO systems before implementation into research and clinical practice. Unlike paper-based PRO, the quality of ePRO data might be impacted by the system’s technical performance, which necessitates developing and conducting a robust test plan. The basis of test planning should be adapted from the technology-related fields of computer science and health informatics to ensure rigorous structure.

Software or system testing is an important phase in the software development life cycle that validates the design quality, functionality, and maintainability. It has been reported that approximately half of the total cost of system development is spent on system testing [14]. Testing is considered a cost-effective procedure as it reduces future time and cost overruns [15]. As in the software development life cycle, system testing is a repetitive and consecutive procedure conducted by the development team and software provider (Table 1). The system provider should test the system prototype once the development team releases it. This form of testing is termed “user acceptance testing (UAT).”

**Table 1:** Definition and roles and responsibilities of user acceptance testing–involved personnel.

Term	Definition	Roles and responsibilities
Development team	Facilitators of the development of a system or electronic system, including system engineers or information technology personnel.	Carry out system designing, coding, delivering, and supporting electronic systems.
ePRO^a^ provider	A person or group accountable for liaising with the development team and implementing ePRO in a designated environment.	Select appropriate PROMs^b^ for electronic conversion. Set business requirements for the development team and perform UAT^c^.
End users	Targeted patients or research participants who are encouraged to use the system and complete the ePRO.	Reported their health outcomes using the ePRO system.

^a^ePRO: electronic patient-reported outcome.

^b^PROMs: patient-reported outcome measures.

^c^UAT: user acceptance testing.

The UAT can be defined as “the degree to which a product or system can be used by specific users to meet their needs to achieve specific goals with effectiveness, efficiency, freedom from risk, and satisfaction in specific contexts of use” [16]. UAT is not supposed to include testing of the system’s internal structure but rather aspects of input and output features (black box testing) [17]. UAT tends to uncover errors that were not possible to detect in the in-house testing [17]. However, the UAT's error detection efficiency completely depends on the system provider’s ability to define a robust plan to efficiently detect errors with test cases that cover all system input and output features. A lack of robust planning may lead to a time-cost burden that may outweigh the benefits of UAT. It should be noted that the UAT described in this section is not the same as “usability testing.” Usability testing aims to investigate the ease and appropriateness of the electronic system from the end user’s perspective. Usability testing is a subsequent step to UAT, which is not in the scope of this paper.

This paper aims to bridge the gap between computer science and health care fields and ensure the validity of newly developed electronic systems designed to capture PRO as data collection tools. To fulfill this aim, the authors proposed a behavior-based model to blueprint the development of robust and unique UAT plans for newly developed ePRO systems. A practical application of a newly developed ePRO system in pediatric dentistry was used to demonstrate the model testing process.

## Methods

### Behavior-Based User Acceptance Testing Model

#### Overview

There are multiple approaches to performing UAT that correspond with the aim of the testing and the nature of the system. A few basic principles are considered cornerstones that must be considered when planning and conducting UAT [18,19]: (1) test cases should not include a member of the development team; (2) each testing phase is dependent on previous successful testing phases; (3) UAT success criteria must not be strictly based on meeting business requirements but rather make sense in a real-world environment; and (4) the quantity of test cases should be based on the context of the system, the end environment, and the manner of usage.

**Figure 1:**
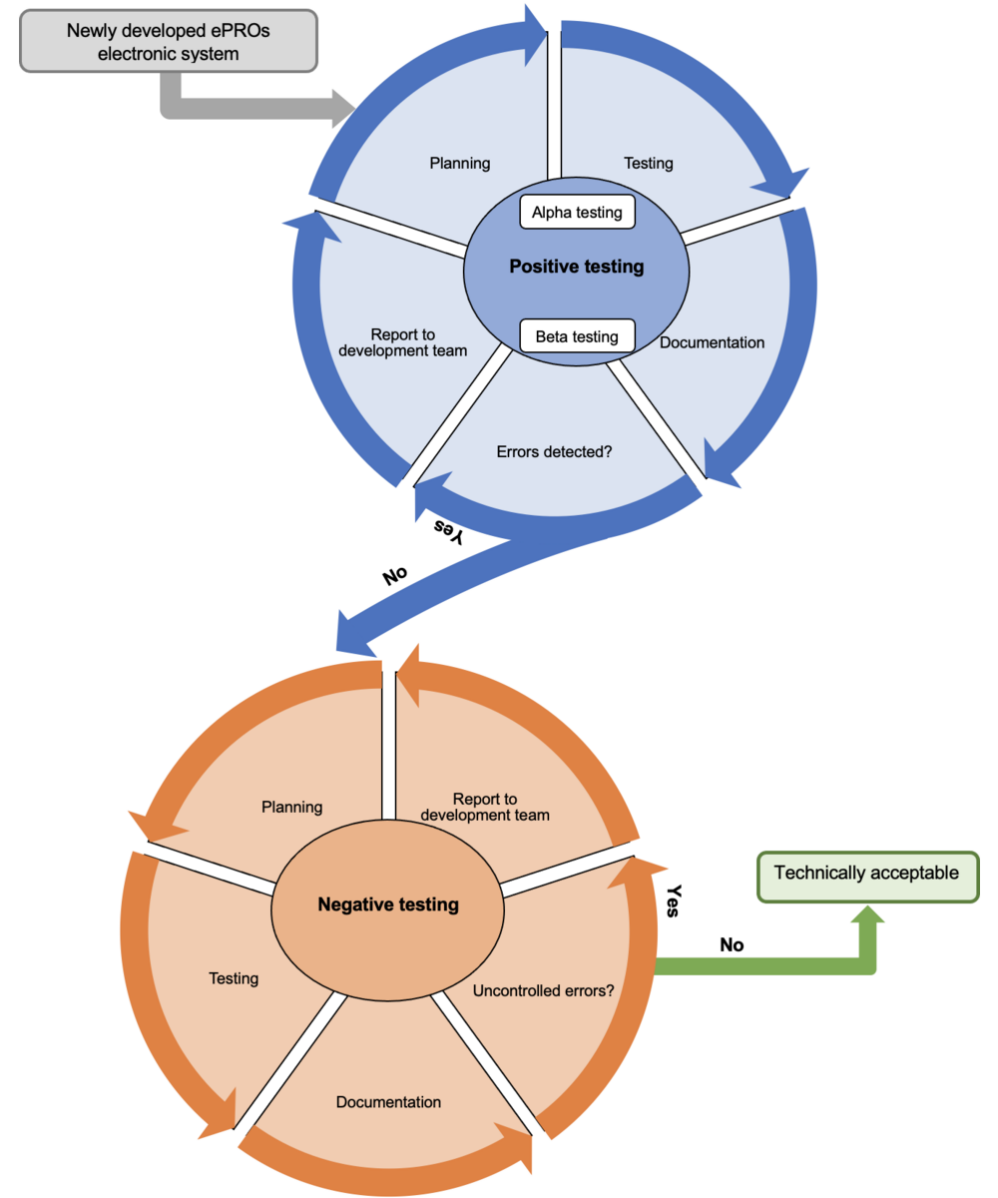
Behavior-based user acceptance testing model for validation of electronic patient-reported outcome (ePRO) systems.

Generally, ePRO is a self-reported measure where patients are expected to remotely complete questionnaires, where their behavior with system specifications is unpredictable and cannot be monitored. The concept of the UAT behavior–based model relies on the fact that electronic systems depend on patient behavior as end users within the defined specifications. Patients either use the ePRO system as intended (positive pathway) or against system specification (negative pathway). Negative use of the ePRO system may occur due to unintentional actions or due to the lack of clear instructions. The proposed model focuses on developing deliberate positive and negative test cycles to inspect the input and output features of the ePRO system (Figure 1). In addition, the model includes testing ePRO in an internal, closed environment (alpha testing) and an external, open environment (beta testing) to ensure resemblance to the real world and control potentially influential factors (Table 2).

**Table 2:** Summary of test components incorporated within the behavior-based user acceptance testing model.

Test type		Purpose	Test cases	Tools required	Success criteria
**Positive testing**					
	Alpha testing	To identify errors and influential factors.	Scenario-based	Checklist	No errors or adverse events were detected.
	Beta testing	To identify errors in a real-world environment.	Patient participants	Checklist	No errors or adverse events were detected.
Negative testing		To determine the system’s ability to correctly address incorrect or inappropriate actions.	Scenario-based	List of negative actions	Errors and adverse events are controlled.

Positive testing refers to test system functions as instructed in a closed environment by the system provider, which is known as “alpha testing,” and in the open environment with participants, which is known as “beta testing.” Negative testing refers to test system function against instructions.

Terms and methods used in this model are adapted from computer science literature [17]. The list of personnel terms and responsibilities is summarized in Table 1. A detailed description of the model components and pathways is discussed as follows:

#### Positive Testing

Positive testing describes a test of the validity of system specifications under responses expected to valid inputs [20]. Positive testing for ePRO should be conducted in 2 phases: alpha and beta testing. Alpha testing is a type of internal acceptance testing conducted primarily by an ePRO provider, whereas beta testing is external testing conducted by a group of external users (ie, patients). Alpha and beta testing are equally important in identifying system errors and potential risks [21]. Positive testing requires a test script or checklist to ensure the thoroughness of the test and lower the risk of omitting features.

#### Positive Testing Checklist

Testing is a meticulous inspection of a product to identify overlooked issues or omitted features based on criteria. It is critical to develop a systematic approach to positive testing to control tester subjectivity and reduce the risk of error omission. Checklists are a common practice and a well-accepted technique to ensure all important system requirements have been considered [22]. The positive testing checklist should be unique and include all requirements and desired outcomes as set by the system providers.

The positive testing checklist can be developed and divided into 2 main aspects based on the end user point of view of input users (participants or patients) and output users (researchers or clinicians). Patients or participants completing ePRO and clinicians or researchers reviewing the final reports are 2 distinct groups with distinct expectations for the performance of electronic systems. Furthermore, the positive testing checklist should be divided into categories and subcategories to ensure that different aspects of the system, such as appearance, content, number, and function of items, are inspected to determine whether the desired outcome is achieved. The addition of free-text boxes in the positive testing checklist is important so that testers can describe undesirable events as they occur and describe errors not covered by the checklist items. It is crucial to pilot the positive testing checklist to ensure thoroughness before conducting alpha and beta testing.

#### Alpha Testing

Alpha testing is the primary form of testing and should be rigorously structured with case-controlled tests aimed at testing ePRO iterations in stimulating technical situations to test system compatibility with different operational elements [23]. Alpha testing requires multiple test cases with fixed external influential factors that may affect the ePRO system’s performance. For example, different electronic devices with different screen sizes, operating systems, types of internet connections, sites of completion, and web browsers. Alpha testing should be conducted before testing the ePRO in an open environment with a group of patients, as the source of errors is difficult to detect.

#### Beta Testing

The term “beta testing” refers to any form of testing performed in an external open environment to evaluate the system’s behavior in real-world scenarios with end users [24]. In this model, beta testing aims to test the ePROM with a small group of patients or participants in a targeted clinical practice environment or a recruitment site. The inclusion of the beta testing element in the UAT model is driven by the acknowledged need to involve patients and the public as service users in health care and health research [25]. Involving patients in the testing process would reveal undetectable errors from the alpha testing and show the actual status of the ePRO system due to different user behaviors and the use of other devices with different configurations. The decision to execute beta testing should be based on the outcome of the alpha testing.

There is no strong evidence or guidance available on the number of participants required to achieve a significant probability of detecting the majority of errors in specific system iterations. ePRO providers can estimate the required sample size based on the complexity of the ePRO system and the number of specifications. Ensuring user diversity by including users from different groups or roles is important for capturing a range of perspectives, user behaviors, and potential issues [26].

After a positive testing cycle is completed, outcomes and detected errors should be reported to the development team. The positive testing cycle should be repeated until the ePRO system reaches an iteration with a stable and error-free performance.

#### Negative Testing

The negative testing principle is the opposite of positive testing, where the normal flow of logic is tested. Negative tests are performed to ensure that the system is able to process and control incorrect or inappropriate responses [27]. In computer science literature, negative testing is an accepted method of assessing the ability of software or a system to detect threats and conflicts and to understand the sources of invalid outputs [28].

The inclusion of negative testing in the proposed model is justified, as patient behavior cannot be monitored when using the ePRO system. Patients either positively react to the ePRO system interface, withdraw from completion, deviate from following instructions, or have a normal intuitive response. Unlike the paper-based method of PRO collection, ePRO is delivered by a system that, if misused, may lead to undetectable errors and, therefore, impact the meaningfulness of PRO data. The necessity to execute negative testing does not identify unexpected behavior and patterns of use but instead increases confidence in the technical performance and security of the ePRO system [28].

The ePRO provider may have a list of possible end user negative actions according to the ePRO system specifications, such as overfilling the free text box, selecting multiple PRO responses per item, inputting dates in a different format, or skipping essential items. The negative test cycle ends when the ePRO system reaches an iteration with controlled unfavorable outcomes that may directly or indirectly impact the quality of collected data.

### Documentation and Outcome Reporting

Documentation of the UAT process is crucial to ensuring efficient testing and progress tracking. In addition, it is a best practice in system development to document testing details and meet the expectations of regulatory bodies [29]. Documentation should be integrated into all the UAT steps, including testing planning, execution, and outcome. Finally, a formal agreement document to sign off on the UAT and to either accept or reject the developed system. ePRO providers may add instruction documents to facilitate a standardized UAT process between multiple testers or centers [13]. To ensure good communication with the development team, the UAT cycle report must be written in simple language with illustrative screen captures of system errors or unwanted features. Once the ePRO system has reached technically acceptable performance, ePRO providers and the development team should sign off on the UAT, and all documents must be archived for future reference, institutional inspection, clinical audits, and publications.

Following a successful implementation of the ePRO system, ePRO providers must provide periodic check-ups and open communication channels with the end user (patients, participants, clinicians, or researchers) to facilitate performance monitoring and incident reporting.

### Application of Behavior-Based Model in Pediatric Dentistry

The proposed UAT model was applied to a newly developed web-based ePRO system designed for routine clinical use in pediatric dentistry. The details of the model application and outcomes are discussed in the following subsections.

#### Electronic Personal Assessment Questionnaire-Paediatric Dentistry

The authors are leading a research project investigating the feasibility and utility of the routine use of ePRO in pediatric dentistry at Charles Clifford Dental Hospital (Sheffield Teaching Hospitals NHS Foundation Trust, Sheffield, United Kingdom). The electronic Personal Assessment Questionnaire (ePAQ [ePAQ Systems Ltd]) platform technology was selected to facilitate the delivery and collection of child oral health ePROs. The ePAQ-Paediatric Dentistry (ePAQ-PD) version was developed following the electronic conversion of the 12-item caries-specific PRO (Caries Impacts and Experiences Questionnaire for Children [CARIES-QC]) and a short-form dental anxiety measure (8-item Children’s Experiences of Dental Anxiety Measure [CEDAM-8]) [30,31]. Additional items were added to the ePAQ-PD, including parental consent, child assent, and free text box items to record comments and ask the dentist questions.

The ePAQ-PD is a newly developed system that has successfully met software engineering requirements. The conduct of UAT was considered to outline the level of technical readiness of ePAQ-PD before introducing the system to routine clinical use. Principles of the behavior-based UAT model were applied as detailed in the previous sections.

#### Application Procedure

The application of the behavior-based model was performed with discussions with the ePAQ clinical lead and software engineers.

For positive testing, the research team developed a checklist for UAT until consensus was achieved. The UAT checklist was designed to cover different aspects and functions of the system (Table 3). The UAT checklist was piloted and showed excellent consistency in reviewing ePAQ-PD functions. Alpha testing was conducted using 4 test cases to imitate different situations with factors that may influence how the ePAQ-PD system might be accessed and completed. Test cases include using different age groups to test skip logic functions and output on the final report. Different technology-based situations were included, such as using different electronic devices, operating systems, email providers, web browsers, internet connections, cable types, wireless and cellular. Test cases also include accessing and completing ePAQ-PD at different times to ensure the performance is stable throughout the day.

**Table 3:** Example of a positive testing checklist for the electronic Personal Assessment Questionnaire-Paediatric Dentistry system.

Feature				Successful or Correct		Failed or Incorrect	Comments	
**Input data**								
	**Access**							
		Provider access to the system						
		Generation of invitation letter						
		Delivery of invitation letter to end user						
		End user access to the system						
	**Responsiveness and content**							
		Number of items						
		Function of response options						
		Function of navigation options						
		Position of response and navigation options						
		Skip logic algorithm						
		Submission of responses						
	**Appearance**							
		Appropriate font type						
		Appropriate font size						
		Appropriate graphics						
		Appropriate color						
**Output data**								
	**Content**							
		Date of completion						
		Participant details						
		Consent and assents						
		Participant responses						
	**Appearance**							
		Appropriate font type						
		Appropriate font size						

Following the completion of alpha testing, children and their parents or caregivers who attended the pediatric dental department were recruited regardless of the reason for attendance in the beta testing stage. Following their verbal approval, a web link to the ePAQ-PD log-in page was generated and sent to the patients by email to their parents or caregivers. Children were asked to complete the ePAQ-PD, and parents were asked to provide assistance if necessary.

Negative testing was conducted on the basis of executing opposite or neglectful actions of inputting wrong data, skipping items, and deleting output data; a list of possible end user negative actions was developed and piloted. Negative testing was repeated until all errors were controlled.

#### Participants

Children and their parents or caregivers attending the Paediatric Dentistry Department at Charles Clifford Dental Hospital were recruited regardless of the reason for their visit. In total, 10 children were purposively targeted to be recruited for beta testing per ePAQ iteration. Children were selected based on age groups (3-8 years, 9-10 years, and 11 years and older) to test the ePAQ-PD skip logic functions.

### Ethical Considerations

This study was approved by the Clinical Effectiveness Unit of Sheffield Teaching Hospitals NHS Foundation Trust as a service evaluation project (project: 11057). Information regarding the ePAQ-PD system, reasons for testing, and their role in the testing process were explained to participants. Electronic child age-appropriate assent forms and parent or caregiver consent forms were completed by participants that were incorporated into the ePAQ-PD system. Participants were not compensated for their time. Participants were anonymized for analysis using unique identification numbers.

## Results

The ePAQ-PD system achieved technically acceptable performance after 3 positive test cycles and 1 cycle of negative testing. Alpha testing was conducted 5 times with 25 test cases. For the beta tests, 30 participants of different age groups and their parents or caregivers were recruited for the 3 cycles of testing. The age range and number of participants recruited in the beta testing stage are shown in Table 4.

**Table 4:** Age range and number of participants recruited in the beta testing stage (n=10 per iteration).

Beta testing		Participants, n
**First iteration (age range in years)**		
	3-8	3
	9-10	2
	11-16	5
	Total	10
**Second** **iteration (age range in years)**		
	3-8	4
	9-10	1
	11-16	5
	Total	10
**Third iteration (age range in years)**		
	3-8	6
	9-10	2
	11-16	2
	Total	10

Several technical errors were found with iterations in both the alpha and beta test cycles. According to the UAT checklist used, 13 errors were detected in the first iteration and 2 errors in the second iteration. Alpha testing was only able to detect 33% (5/15) of the total errors, while beta testing detected the remaining 67% (10/15) of errors. Errors were related to system failure to produce correct scoring of ePROMs, final patient reports, and reminder invitation emails. The third iteration of the ePAQ-PD system showed no technical errors and was selected for negative testing.

The majority of negative actions applied to the third iteration of the ePAQ-PD system showed favorable responses. The current iteration showed only 1 unfavorable response to negative actions, allowing the user to skip the age range item. By default, the ePAQ-PD system assumes an end user age range between 3 and 8 years upon skipping this item. This response is considered unfavorable as it may lead the ePAQ-PD system to produce incomplete results. The error related to skipping the age range item was discussed with the development team, and a decision was made to prevent the user from skipping this item.

## Discussion

This study managed to bridge the gap between the 2 fields of computer science and health care. A behavior-based model of ePRO system validation was developed based on the principles of UAT and patient-centered care. The proposed model showed broad conceptual pathways that ePRO providers may consider when planning the validation of electronic systems. The application of the model to a newly developed ePAQ-PD system showed a high ability for technical error detection in a systematic fashion. The ePAQ-PD system achieved technically acceptable performance after 3 positive test cycles and 1 cycle with negative testing.

The proposed model allows systematic inspection of system specifications and identification of technical errors through simulated positive and negative usage pathways in open and closed environments. It has a generic structure to ensure its applicability to different PROM data acquisition systems. Contemporary literature lacks technical testing forms for electronic systems designed to collect PROM data, which reflects the novelty of the model and the area being investigated. It is anticipated that the development and application of the behavior-based model will inspire researchers to draw attention to the importance of technical testing of ePROM systems and the development of further models. The practical application of the model to the ePAQ-PD system showed a few points that demonstrate the model's thoroughness and robust structure. In general, the high number of errors detected with the first iteration reflects the necessity of the technical testing of the ePRO system before implementation. Beta testing revealed more errors than alpha testing, which supports the notion of the behavior-driven concept of the proposed model. Negative testing revealed unfavorable responses that would be difficult to detect if the ePAQ-PD system was implemented in clinical practice.

A few limitations within the proposed model must be noted to ensure appropriate application in future work. The behavior-based model has limited flexibility and does not account for any forms of alteration or addition to the electronic system during testing. The model failed to include researchers or clinicians as output end users in beta testing, where their inclusion may reveal additional undetectable errors in system management features and final reports. The exclusion of output end users in this model was driven by a cost-effectiveness assumption, as unlike patients or participants where previous knowledge or experience is not required, researchers or clinicians require training to access the system management dashboard and view final reports. The provision of training sessions would create an unnecessary burden for the testing system prototype, where ePRO providers can act on their behalf. The proposed model requires a defined postimplementation monitoring plan and a long-term maintenance strategy to identify and address any issues that may arise after the system is implemented.

In conclusion, a behavior-based model with a generic structure has been developed to ensure its applicability in testing different PROM data acquisition systems. The proposed model has increased the confidence in the validity of ePAQ-PD as an electronic system. Further application of the behavior-based model in future studies is required to fully ascertain efficacy.

## Abbreviations

CARIES-QC: Caries Impacts and Experiences Questionnaire for Children
CEDAM-8: 8-item Children’s Experiences of Dental Anxiety Measure
ePAQ: electronic Personal Assessment Questionnaire
ePAQ-PD: electronic Personal Assessment Questionnaire-Paediatric Dentistry
ePRO: electronic patient-reported outcome
PRO: patient-reported outcome
PROMs: patient-reported outcome measures
UAT: user acceptance testing

## Data Availability

All data generated or analyzed during this study are included in this published paper.
